# Active Monitoring of Residential Radon in Rome: A Pilot Study

**DOI:** 10.3390/ijerph192113917

**Published:** 2022-10-26

**Authors:** Gaia Soldati, Maria Grazia Ciaccio, Antonio Piersanti, Valentina Cannelli, Gianfranco Galli

**Affiliations:** Sezione di Roma 1, Istituto Nazionale di Geofisica e Vulcanologia, Via di Vigna Murata 605, 00143 Roma, Italy

**Keywords:** indoor radon concentration, active radon monitor, residential radon, risk assessment

## Abstract

We present an overview of the potential of active monitoring techniques to investigate the many factors affecting the concentration of radon in houses. We conducted two experiments measuring radon concentration in 25 apartments in Rome and suburban areas for two weeks and in three apartments in the historic center for several months. The reference levels of 300 and 100 Bq/m^3^ are overcome in 17% and 60% of the cases, respectively, and these percentages rise to 20% and 76% for average overnight radon (more relevant for residents’ exposure). Active detectors allowed us to identify seasonal radon fluctuations, dependent on indoor-to-outdoor temperature, and how radon travels from the ground to upper floors. High levels of radon are not limited to the lowest floors when the use of heating and ventilation produces massive convection of air. Lifestyle habits also reflect in the different values of gas concentration measured on different floors of the same building or in distinct rooms of the same apartment, which cannot be ascribed to the characteristics of the premises. However, the finding that high residential radon levels tend to concentrate in the historic center proves the influence of factors such as building age, construction materials, and geogenic radon.

## 1. Introduction

Characteristic of the volcanic soils is the presence of uranium [1] and, therefore, the emission of radon (Rn-222), a noble colorless, odorless, radioactive gas produced by the decay of uranium naturally present in the Earth’s crust. Outdoor radon levels are usually low since radon disperses in the air very fast. However, it may easily enter the buildings either through foundation walls or from construction materials, accumulating mainly in the lowest and least ventilated closed environments. There, concentrations can easily exceed 1000 Bq/m^3^ with important repercussions on health. For this reason, in recent years, the analysis of indoor radon has grown in importance in terms of socio-sanitary issues, especially related to the carcinogenic effects of this gas. The World Health Organization (WHO), the National Academy of Sciences, the US Department of Health and Human Services, as well as the United States Environmental Protection Agency (EPA) have classified radon as a major human carcinogen [2,3] because of the wealth of biological and epidemiological evidence showing the connection between exposure to radon and lung cancer in humans. The buildings more likely to expose the population to a radon-related risk to health are those based on soils of volcanic origin and/or highly permeable [4] and which use construction materials such as tuff, pozzolana, and granite [5]. The extensive use of volcanic rocks as a building material in central Italy makes the population in this area particularly prone to high exposure to this air pollutant [6,7]. Since exposure to high residential radon is a serious public health problem, there are laws setting the maximum permissible concentrations of radon in workplaces and residential dwellings (homes).

The vast majority of the available studies of residential radon activity concentration are based on passive monitoring techniques [8], usually characterized by a single or a few measurements, with sample time ranging from 24–48 h (active carbon) to a few weeks/months (sensitive films/track etches) and resulting in radon concentration values averaged over the sampling time (typical is the evaluation of the annual mean concentration). Examples are the national indoor radon surveys of the European countries (e.g., Italy [9], France [10], Switzerland [11], United Kingdom [12]) and the several monitoring campaigns conducted on a regional scale [13,14,15]. Among the few studies of residential radon employing active devices, we can mention the indoor radon surveys of Cyprus [16,17], the only exception in Europe National campaigns to the passive measurements’ technique and the monitoring of houses in a volcanic environment [18].

The widespread use of passive devices is due to recognized advantages such as low-cost and high nominal accuracy; a problem of evaluating the annual indoor radon concentration on the basis of short-term passive measurements is the need to use temporal correction factors in order to correct diurnal and seasonal variation [19]. Conversely, medium/long-term passive monitoring techniques could suffer from saturation issues and cannot account for potentially important multi-scale temporal variation in radon concentrations. Indeed, in the last decade, several analyses of indoor and outdoor radon time series [20,21,22,23,24] have definitely demonstrated that radon concentrations exhibit marked daily to seasonal variability that can hardly be detected by passive monitoring techniques [19,25]. The capability of fully evidencing such variabilities is a key factor in understanding the dynamics of radon diffusion in indoor environments. Moreover, diurnal and semidiurnal variabilities can be immediately exploited for prevention and mitigation measures since they can be directly related to the human presence in the dwellings. Therefore, in order to implement successful management of indoor air quality, the spotlight is shifting toward continuous monitoring of radon levels in living spaces. With this aim, we conducted two experiments to investigate residential radon levels and their temporal fluctuations.

The first one originated from a governance project (Alternanza Scuola-Lavoro, d.lgs. 77/2005) for the introduction of work themes to undergraduate students. A whole class of students of the Liceo Scientifico Cavour in Rome had the opportunity to measure radon indoors in their houses and to analyze potential links with the geology of the area and housing typologies [26].

The identification of anomalously high values of radon concentration in a house located on the VI floor motivated us to carry out another set of measurements in two apartments of that same building and one belonging to a nearby building (about 300 m apart). Both the premises are located in the Esquiline district, born on the largest of the seven hills of Rome after the enormous building expansion following 1871 when the city became the Italian capital. Still, in 1870, Rome appeared as a small rural city dotted with monumental ruins of antiquity and vast areas of urban gardens and was enclosed within the Aurelian Walls (historic center). This urban development has led to strong differences between areas of recent and historical urbanization in terms of construction techniques and characteristics of the subsoil and of the landfill, which affects specific hazards such as landslides, subsidence, and radon emission. That’s why the Esquilino area is particularly interesting when dealing with residential radon.

The analysis of the two datasets collected during the experiments constitutes the bulk of this study. It is described in the next section one experiment at a time, given the differences in the way we collected the relative time series (number of measurement points, length of recording, season of recordings). The findings of the two experiments are then summarized as general considerations in the final sections.

## 2. Materials and Method

### 2.1. Geological Setting

**Figure 1:**
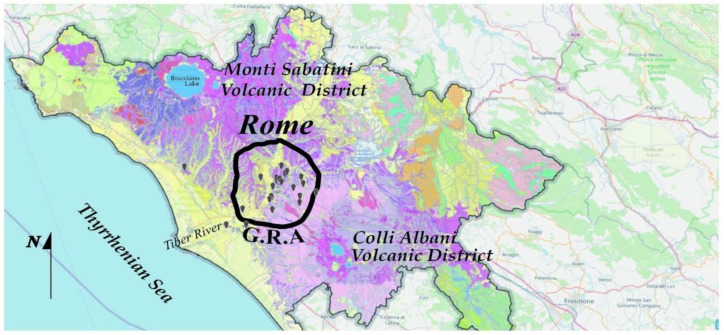
Geologic map of Rome and municipalities of the metropolitan area; the legend is provided at https://g3w-suite.cittametropolitanaroma.it/it/map/dati-territoriali/qdjango/66/ (accessed on 25 July 2022). Deposits from Monti Sabatini Volcanic District crop out north of Rome, and deposits from Colli Albani Volcanic District crop out south and east of the city. Gray pins refer to the houses monitored in our experiments; the black circle represents the “Grande Raccordo Anulare”, the ring-shaped motorway that encircles Rome.

The millenary history of Rome is closely connected to its peculiar geological history. In its territory, soil outcroppings represent products of both volcanic and exogenous activity. The volcanic district of the Colli Albani (20 km SE of Rome) and that of the Mti Sabatini (NW) determined the deposition of a thick pyroclastic blanket, creating a vast plateau [27]. The eruptions of the Colli Albani volcanic complex, in particular, have led to the placement of numerous and extensive pyroclastic formations, which as a whole cover most of the territory of the Municipality of Rome and which develop mostly in the east and south of the city [28]. These pyroclastic products are mainly constituted by the so-called “ancient tuffs”, the complex of the lower pozzolana, the tufo lionato, the upper pozzolana, and the tuff of Villa Senni [29]. The morphology of the territory of Rome is influenced both by natural phenomena such as these volcanic deposits and the alluvial deposits of the watercourses that cross it. Figure 1 shows the geological map of the Lazio region, where the ring-shaped highway around Rome is indicated by the thick black line, and the gray pins represent the measurement points of our experiments.

The use of volcanic rocks as a building material in central Italy has been reported throughout centuries, from the Etruscan civilization until the present; in particular, ancient Rome has been built mostly with volcanic rocks from the Sabatini volcanic District [30,31]. The extensive use of tuffs as a building material is due to their intrinsic properties [32]. In fact, tuffs are easy to cut and fit into blocks of almost any shape and size; they are resistant to weathering and, at the same time, have densities lower than most other stones; moreover, tuffs are also efficient thermal insulators. However, tuffs are known for their radioactivity, usually higher than other rocks, leading to higher exposures for the population in this part of Italy to major health risks [6,7].

### 2.2. Instrumentation

We adopted small-sized commercial solid-state radon detectors produced by the company Algade, model “AER C”, specifically modified and calibrated for use in the Italian Radon Monitoring Network [33]. The establishment of precise and accurate equipment calibration protocols is, in fact, a critical factor in radon measurement, especially when the data collected by several instruments have to be compared. To meet the internationally recognized standards, a radon chamber available at the INGV Radionuclide laboratory has been used to verify, calibrate, and characterize the instruments for the measure of both soil gas and indoor radon activity concentration.

Since certification of their robustness to variable environmental conditions is necessary, the influence of water content in the detection volume, which may affect the solid-state detectors installed in the instruments, must be evaluated. We have studied the response of every radon monitor by varying the degree of humidity to which it can be exposed; as a result, a customized correction function has been derived for each sensor to ensure data correctness in all operating conditions [34].

After applying the above procedures, these low-cost radon detectors originally designed for consumer applications exhibit performances comparable with those granted by devices specifically designed for scientific applications. The range of ^222^Rn values measurable by the detectors varies from 0 to tens of thousands Bq/m^3^, with a sensitivity of the order of 20 Bq/m^3^ for each pulse/h. The measurement error at 300 Bq/m^3^ is approximately 5% over a daily measurement interval. The instruments operate with environmental temperature values between 0 and 40 °C and at relative humidity preferentially <80%; however, its correct operation has been verified even at humidity values close to 100%. The instruments, in addition to radon concentration, simultaneously acquire local temperature and relative humidity. The acquisition (sampling rate) is set to 4 h. Based on the probability, at a 95% confidence level, that the instrument provides a non-zero concentration value, the estimated detection limit for the 4-h measurement is 15 Bq/m^3^.

AER radon detectors are not susceptible to thoron, which could lead to unreliable and overestimated values of radon concentration because the setting of the energetic window enabled for counting is optimized for detecting Po-218, and the gas enters the instrument through a barrier diffusion.

### 2.3. Study Design

The dataset has been collected as a result of two experiments, covering different time periods conducted in the city of Rome and the suburban area.

In Experiment 1, 4 instruments were placed in turns/alternatively in 25 apartments for about two weeks, during the period December 2017–April 2018. The sampling time has been set to 4 h, except in 3 apartments (DanielS, LudovicoJ, and ValerioA), where it was set to 2 h. Due to the different permanence times of the detectors in the various apartments, the time series have different lengths, ranging from a minimum of 9 to a maximum of 47 days.

In Experiment 2, 3 apartments housed 5 radon detectors, 2 of which were in 2 different rooms of the same apartment, throughout the period ranging between February and October 2021. The resulting time series have different lengths because only 3 instruments kept working for the whole scheduled duration of the experiment. The collected radon concentration data show a few peaks or abrupt changes from the average radon level that we deem as instrumental anomalies. Indeed, radon detectors for active monitoring are known to be influenced by electromagnetic perturbations [34], which in some cases may result in spurious data that we manually removed from the radon time series. Having verified that for residential indoor measurements, the impact of the specific correction curves for temperature and relative humidity (T-RH) is limited, in the present analysis, we have considered the raw data [34].

## 3. Results

### 3.1. Experiment 1: ASL Liceo Cavour 2017

**Figure 2:**
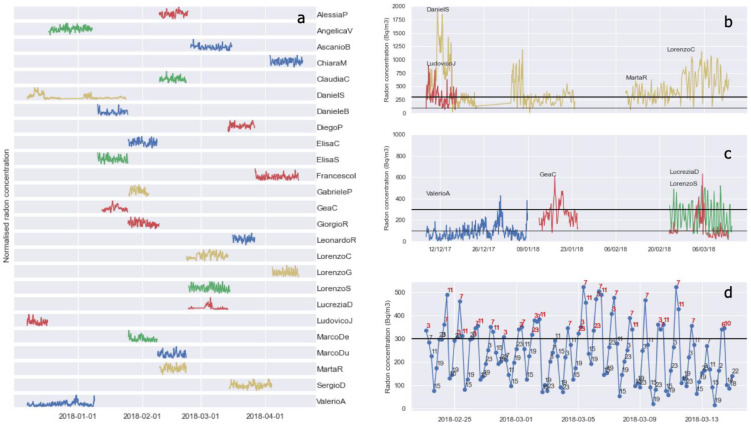
(**a**) Time series of normalized radon concentration recorded in 25 apartments. The four instruments employed (indicated by colors red, green, blue, and yellow) remained in each location for about two weeks, over the interval December 2017–April 2018. (**b**) Time series of radon concentration with average radon exceeding the EURATOM—recommended threshold of 300 Bq/m^3^ (thick black line). The WHO—recommended threshold of 100 Bq/m^3^ is shown as a thin black line). (**c**) Time series with average radon level below the threshold of 300 Bq/m^3^ and maximum radon level exceeding it. (**d**) Radon time series measured in LorenzoS apartment. The hour of recording is indicated for each single radon data, with red labels indicating values exceeding 300 Bq/m^3^.

The radon time series that the 25 students involved in the experiment recorded in their homes are shown in Figure 2a. They are named after the students themselves and normalized to the maximum radon value. The four instruments employed are indicated by different colors; note that some measurements lasted more or less than the scheduled recording period of 2 weeks due to some difficulties in the exchange of the radonometers from one student to the other or to the need for investigating more deeply the houses with radon levels higher than expected (e.g., DanielS).

To assess the extent of the indoor radon concentrations found, we refer to the Euratom Directive 2013/59 [35], adopted in Italy with D.Lgs. 101 of 31/7/2020, which recommends for workplaces and public buildings a maximum annual average concentration value for indoor radon air in 300 Bq/m^3^, and to the advice of WHO [2], which established, on the basis of purely sanitary issues, a reference level of 100 Bq/m^3^. These thresholds cannot be directly compared to the mean radon concentration measured on short time intervals [36]; however, they can serve as terms of comparison considering that since data collected in the cold season (heating season) provide the highest values of the indoor radon, the seasonal corrections applied in order to make the results valid for the whole year can be omitted if the conservative approach is applied.

**Figure 3:**
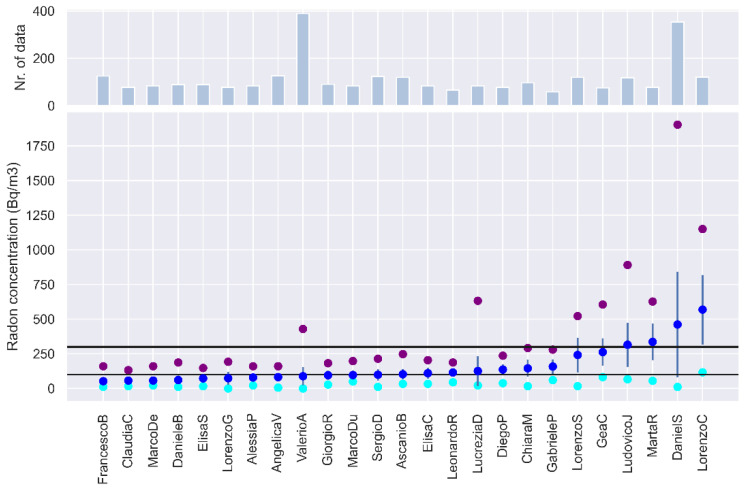
Mean (blue), minimum (cyan), and maximum (purple) values of radon concentration (Bq/m^3^) for the time series recorded in the 25 apartments of Experiment 1. Error bars represent the standard deviation, and bars in the top panel indicate the number of collected data.

Figure 3 illustrates the descriptive statistics of the collected time series: along with mean, minimum, and maximum values (blue, cyan, and purple circles, respectively) of radon concentration, the standard deviation (blue error bars), and the number of data of each time series (top panel) are included. It can be observed that 52% of the apartments (13 out of 25) are considered present an average radon level above 100 Bq/m^3^, and 16% (4 out of 25, Figure 2b) also exceed 300 Bq/m^3^. Even when the average radon level measured in the apartments is below the reference level, the maximum values recorded sometimes exceed it for much of the day or night, as happens for the four apartments of Figure 2c. The temporal variations of radon concentration observed inside the apartment of LorenzoS (Figure 2c, in green) are illustrated in more detail in Figure 2d, where every radon observation is labeled with its recording hour. It is evident that the highest radon concentration is found between 11 pm and 11 am, a part of the day when people are more likely to be at home.

This suggests that passive instruments commonly used for environmental monitoring may not be the most suitable for detecting all the potential radon-related dangerous conditions in houses due to daily fluctuations of radon gas. In fact, this type of instrument provides an estimate of the average concentration of radon over time which, as demonstrated in these experiments, is often below the threshold and is not able to fully evidence high values potentially representing a safety issue (like for example, persistent high radon levels found during the night).

The indoor radon level measured at nighttime is, in fact, a critical issue not only for the more likely presence of people at home in this part of the day but also because radon concentration is usually higher at night. This happens because of the larger temperature gradient between the inside and the outside, especially during the cold season.

**Figure 4:**
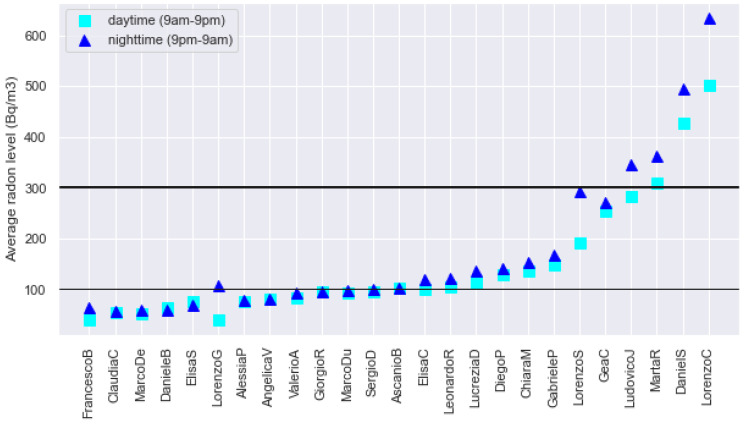
Average radon concentration computed during daytime (light blue squares) and nighttime (blue triangles) in the 25 apartments of Experiment 1. The EURATOM—recommended threshold of 300 Bq/m^3^ and the WHO—recommended threshold of 100 Bq/m^3^ are indicated as thick black, and thin lines, respectively.

To address this question, we split the whole day into two intervals: “nighttime” (from 9 pm to 9 am) and “daytime” (from 9 am to 9 pm), and plot in Figure 4 the average radon concentration computed over each of them. The threshold of 100 Bq/m^3^ at night is overcome in 15 out of 25 apartments (60% of the total), while four apartments (16% of the total) have average overnight radon exceeding 300 Bq/m^3^. A sensitivity test performed by shifting the “nighttime” interval of 2 h both back and forth shows that the impact of changing the definition of this interval is non-significant since the above results vary at maximum by 4%. Notice that considering the maximum values of night radon, independently of the definition used, they overcome 300 Bq/m^3^ in 32% of the cases and 100 Bq/m^3^ in even 100% of the cases.

Overall, most of the apartments (92%) have higher average radon during the night than during the day, although just a few of them with large discrepancies. Remarkably, the largest differences (both absolute and relative) between radon values observed at daytime and nighttime correspond to the apartments where the average radon level is higher, so the active monitoring results are even more effective where more pronounced is the health risk for the inhabitants of the houses. Since, incidentally, our measurements were conducted during the cold season, when the heating systems are switched on, these results are obviously affected by the active convection of air/gas induced by house heating, whose impact we are not able yet to quantify fully.

**Figure 5:**
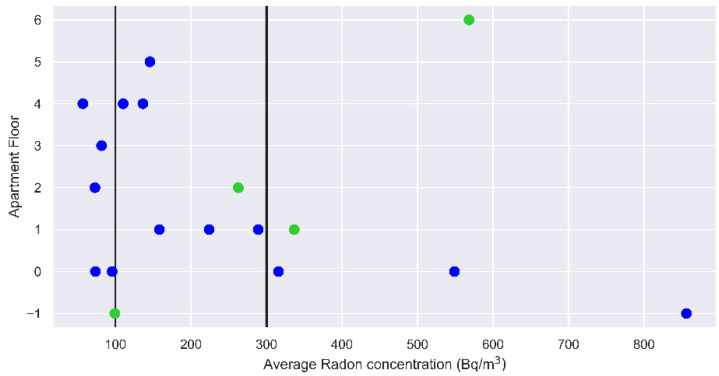
Average radon concentration versus the apartment floor (when this piece of information is available). Green circles refer to apartments in buildings located in Rome’s historical center, built between 1870 and 1900. Black vertical lines mark the reference levels recommended by EURATOM (thick) and WHO (thin).

It is a common belief that the risk posed by indoor radon is larger on the lowest floors of buildings than on the highest ones. This is motivated by the fact that, when entering a building, radon gas mainly accumulates in the lowest spaces, such as the basement or the ground floor, because it mostly penetrates through the foundation walls. We have plotted in Figure 5 the floor of the apartments versus the average radon concentration measured. Even if the figure refers to a subset of the apartments monitored in Experiment 1 (the ones for which such information was available), it clearly indicates that houses with elevated floors do not guarantee safe levels of indoor radon. In fact, we found high radon levels on the sixth floor and radon concentrations between 200 and 400 Bq/m^3^ on the intermediate floors. For smaller radon concentrations, the floor of the apartment appears to be rather irrelevant. On the other hand, we found apartments on the ground floor of buildings based on tuff (e.g., SergioD) where the indoor radon level does not exceed the WHO threshold, suggesting that good construction and insulation from the ground could lead to a reduction in the indoor radon level, and mitigation of the risk for the health of residents.

**Figure 6:**
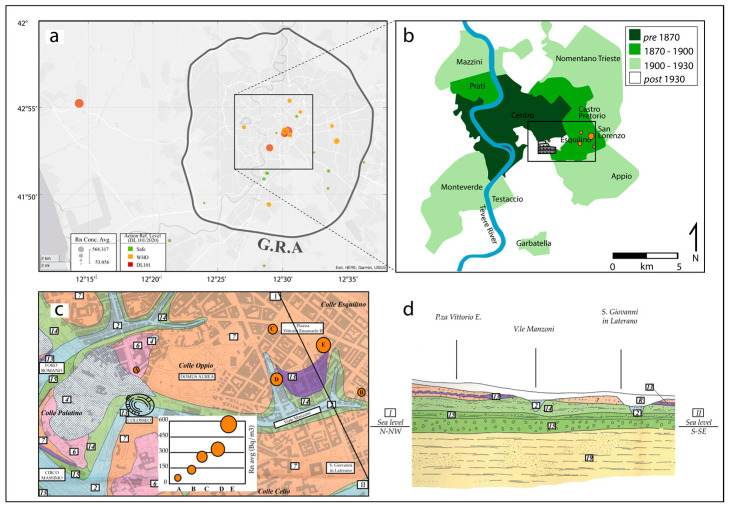
(**a**) Map of average radon concentration (Bq/m^3^) recorded in the 25 apartments of Experiment 1. The size of the bubbles scales with average radon concentration (Rn Avg). Colors represent the category of risk to health: green stands for SAFE values (RnAvg < 100 Bq/m^3^), orange stands for values under WHO threshold (RnAvg < 300 Bq/m^3^), red stands for values over EURATOM threshold (RnAvg > 300 Bq/m^3^). (**b**) Urban development in an area of the city of Rome corresponding to the square inset of Figure 6a; colors indicate the age of buildings (modified from [37]). (**c**) Location of the apartments (orange circles) of the historic center on the geologic map of Rome (modified from [38]). Legend of the geologic units: R Riporti (Anthropogenic deposits); 2 Recent alluviumi; 4 Fluvio lacustrine deposits; 6 Tufo Lionato; 7 Pyroclastic fall; 13 Tufo del Palatino; 14 Paleotiber b; 15 Paleotiber a; 19 Mt Vaticano. Bubbles labeled A, B, C, D, and E refer respectively to apartments FrancescoB, SergioD, GeaC, MartaR, LorenzoC. (**d**) I-II Trace of section; section Scale: Lengths 1:10,000, Heights 1:2000 (modified from [38]).

To visualize on a map the findings described in Figure 2 and Figure 3, geographical distribution of the average values of indoor radon concentration observed in Experiment 1 (represented by the size of the bubbles) is given in Figure 6 12 out of 25 houses (48%) present an average radon level below the WHO threshold of 100 Bq/m^3^ (green bubbles) and can reasonably be considered at low risk. The national reference level of 300 Bq/m^3^ established by Euratom is overcome in four houses (16%, red bubbles), which should need active prevention measures in order to reduce it. The remaining 36% of the apartments (orange bubbles), with average radon between the two thresholds, should be monitored with particular attention. Notably, since the experiment was conducted in winter and spring, when radon indoors is generally higher than during the warmer season, the values of radon concentration found should be considered as superior limits.

The relatively low number of measurement points and their uneven spatial distribution does not allow to draw robust general conclusions; however, the map in Figure 6a shows a significant cluster of apartments with high indoor radon in the historic center of Rome, where only a couple of green bubbles (”safe” houses) are present. Since the building evolution of Rome is characterized by old constructions downtown and progressively younger buildings moving towards suburbia (corresponding to the expansion of urbanization, as illustrated in Figure 6b), this might indicate the influence on the measured radon concentration of the buildings’ age, which, in turns, is related to the type of construction material employed (the older the building, the larger the amount of tuff used).

One final aspect to consider in discussing the dataset of Experiment 1 is the impact of the soil geology on the radon level measured in the apartments. Indoor radon is, in fact, controlled by both anthropogenic and natural factors. Natural (or geogenic) factors are related to radon generation and transport in the ground and depend on geology and other soil properties [39]. Since geogenic radon is an important predictor of indoor radon [40], we investigate the relationship between indoor radon measured in the buildings and deposits upon which they are based. In Figure 6c, the location of the apartments of the historic center (orange bubbles with size proportional to the average radon level) is superimposed on the geological map of Rome [37]. Most of these properties (4 out of 5) have a foundation in volcanic deposits, and the house of MartaR lays on a thin fluvial deposit overlying a volcanic one, as shown in the geological section [37] of Figure 6d. However, for the same deposit, we observe quite different radon values among different houses (lower radon in FrancescoB and SergioD compared to the others) and also for the same building typology (as is the case for GeaC and LorenzoC), suggesting that other factors (related to building characteristics or habits of residents) have a stronger impact than the soil radon emissions.

### 3.2. Experiment 2: Esquilino 2021

Experiment 2 originated from the unexpectedly high radon concentration found during Experiment 1 in an apartment (LorenzoC) on the VI floor. To further investigate this case, two detectors were placed in different rooms of LorenzoC flat (CV6SA, CV6SB), and two more (CV1SA, CV1SB) in different rooms of an apartment on the I floor of the same building. The name CV6SA should be intended as CV-6-SA, where “CV” denotes the building’s address (via Conte Verde), “6” indicates the floor, and “SA” stands for Station A; likewise, CV1SA refers to the building “CV,” floor 1, Station A, and so on. Then an additional instrument was installed inside the apartment of GeaC (GEA) on the II floor of a nearby building. Both the premises belong to the Esquilino district, in the historic center of Rome, where typical buildings are 5–6 floors high, with vertical walls generally made of tuff masonry with brick appurtenances.

**Figure 7:**
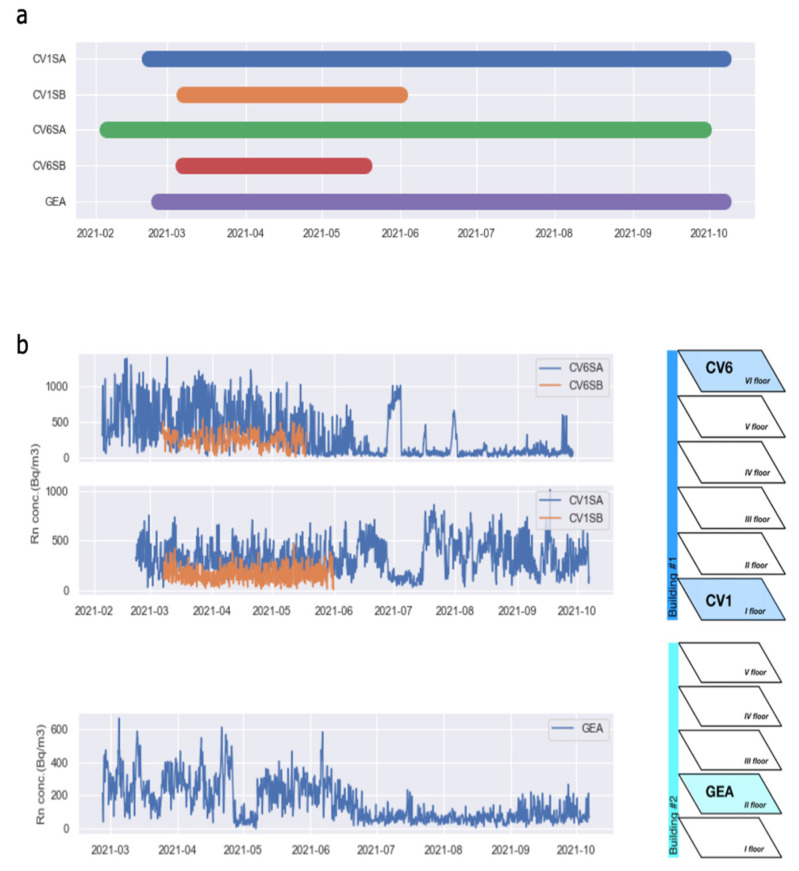
(**a**) Actual recording intervals of the five instruments. (**b**) Radon concentration data was recorded at the five stations considered in Experiment 2. CV6SA-B located on the VI floor of building #1, CV1SA-B on the I floor of the same building, and GEA on the II floor of building #2, according to the sketch on the right.

The plan of Experiment 2 was the active monitoring of five rooms belonging to three apartments for a period of 8 months, from February to October 2021. Unfortunately, instruments located in CV1SB and CV6SB stopped working two months after installation due to power issues (dead battery), so we were left with one instrument for each apartment during June–October, for a total of three instruments working (Figure 7a). The descriptive statistics of the collected time series are reported in Table 1, where the values refer to the time interval March–May 2021, when all the radon devices were operating simultaneously.

Figure 7b shows the radon concentration data measured in the five rooms considered in Experiment 2. The top panels refer to building #1: rooms CV6SA/B are both located on the VI floor, while CV1SA/B are on the I floor. The bottom panel refers to the room GEA in building #2, II floor. As observed in Experiment 1, the average radon level apparently does not reflect the apartment’s floor in the building: while we would expect the highest radon at the lowest floors, the largest values are observed at the VI floor (CV6SA). This result points towards a major role played by anthropogenic factors, such as the use of heating and ventilation, producing massive internal convection, as also suggested by the very different radon levels observed in different rooms of the same apartment (e.g., compare CV6SA with CV6SB, or CV1SA with CV1SB).

The seasonal behavior we expect to observe on the longest radon time series is more pronounced at the high floors due to the generally larger gradient of temperature between the inside and the outside of the house. Anyway, the pressure gradient induced by the temperature gradient has a linear dependence on the elevation of the floor. Figure 7b shows that radon concentration in rooms CV6SA and GEA is higher during the cold season than during the warm one, while in CV1SA, the absolute values of radon concentration do not appear very season dependent. However, the marked frequency variation that can be appreciated in the curve around mid-June might indicate that the influence of season reflects more in the frequency content than in the amplitude of the signal.

**Figure 8:**
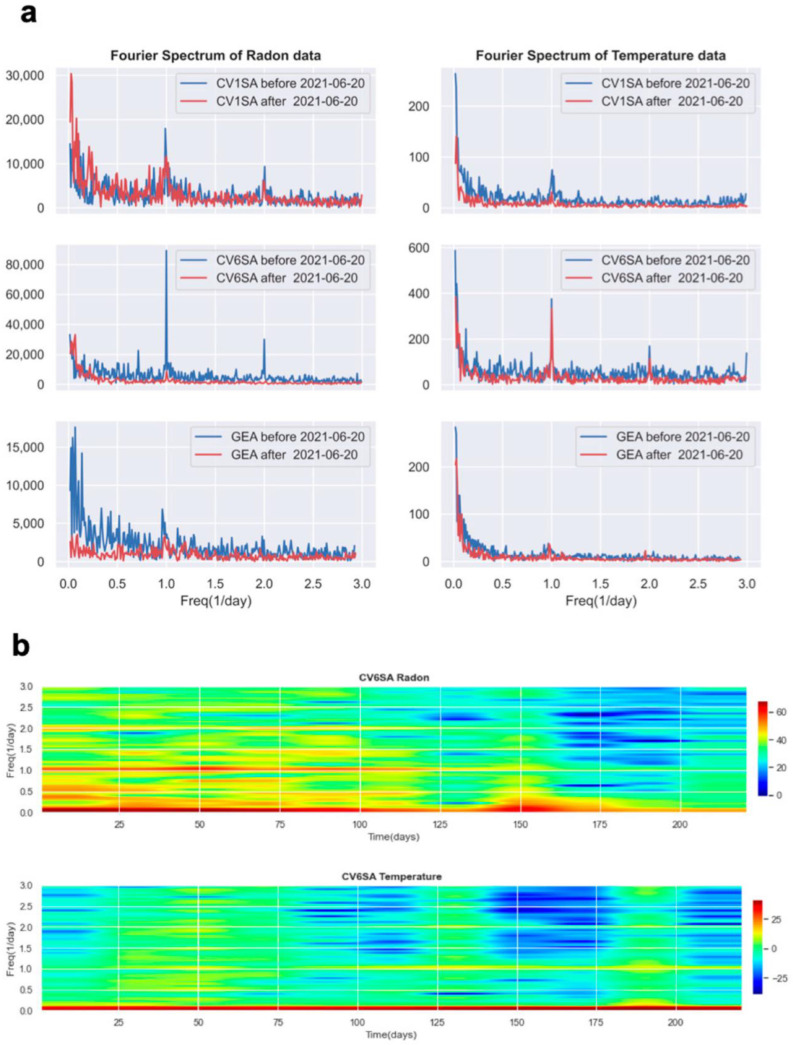
(**a**) Amplitude spectra of the three longest time series of radon (left panels) and temperature (right panels). Blue and red curves refer to data recorded before and after the 20th of June when radon in CV1SA shows a slight change in frequency (Figure 7b). (**b**) Spectrogram of radon concentration and temperature recorded in room CV6SA. Time is measured in days starting from the first day of recording (5 February 2021).

Figure 8a shows the amplitude spectra of the three longest time series of radon concentration (left panels) and internal temperature (right panels). Blue and red curves refer to data recorded before and after the 20th of June when an apparently marked change in frequency appears (see the previous figure). Diurnal and semidiurnal cycles are the most evident feature, and a comparison of left and right plots indicates that the frequency content of radon time series is correlated with the one of temperature. The amplitude of spectra depends on the convection mechanism of radon gas in the dwellings. During the warm season, the temperature gradients among soil, external air temperature, and indoor temperature decrease resulting in a less efficient mechanism of gas radon pumping from the ground to the high floors and in a less pronounced alternate day/night. The peak of period 1 day observed in the spectra of radon in room GEA is evident even if the corresponding peak in temperature is barely visible. This, again, may suggest that gas convection has a non-negligible role in addition to temperature, also in cases such as this one, where the heating has a strong impact (such that radon at daytime results higher than at nighttime, as we will see in the following).

The spectrogram of radon and temperature time series recorded in room CV6SA is shown in Figure 8b, where the *x*-axis represents the number of days since the first day of measurement (5 February 2021). The frequency content of the radon time series is characterized by a strong amplitude of the diurnal cycle at least up to day 125 (mid-May 2021), with the semidiurnal cycle only slightly weaker. Here is even more evident the change in frequency distribution described above, coincident with the beginning of the warm season. The spectrogram of the temperature time series looks weaker than the radon one, yet the 1-day cycle is clearly visible and stronger during the cold season with respect to the warm one.

**Figure 9:**
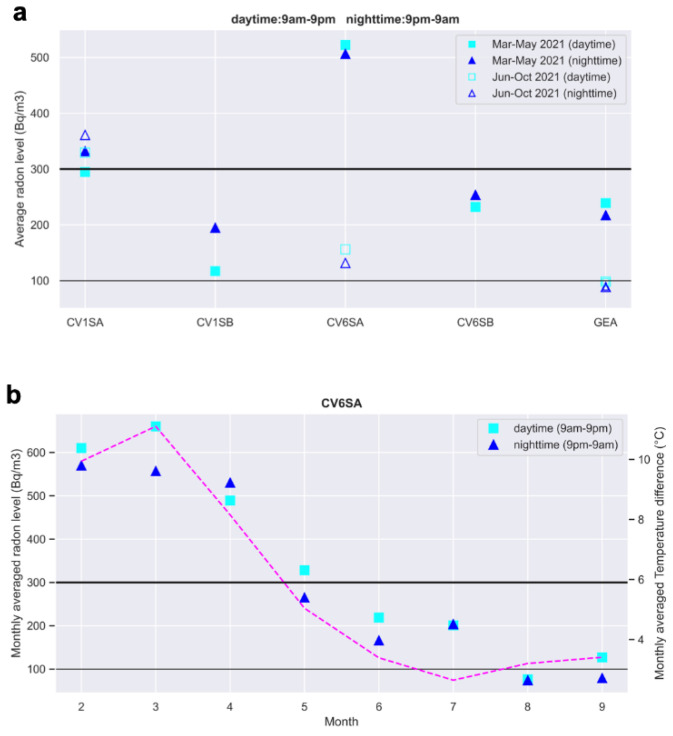
(**a**) Average radon concentration computed over the day and night intervals during the month’s March–May 2021 (5 instruments) and June–October 2021, when only three instruments kept working. (**b**) Monthly averaged radon concentration at station CV6SA computed over the day and night intervals. The dashed line indicates the monthly average of the indoor-outdoor temperature difference. Black lines refer to the reference levels of 300 (thick) and 100 (thin) Bq/m^3^.

During the period of March–May, in part of which the heating systems of the houses are still turned on, we observe higher radon concentration during the night than during the day in 3 out of 5 rooms (Figure 9a, filled symbols). The exceptions are GEA and CV6SA, where daytime and nighttime values are still very similar. In the warm season (Figure 9a, empty symbols), both daytime and nighttime radon decrease significantly in these two rooms, while in CV1SA, the trend is the opposite, with a slight increase in the daytime radon concentration, which overcomes the Euratom threshold of 300 Bq/m^3^. We will see at the end of this section that this is likely an effect of the change in radon transport occurring at the beginning of June: the damping of the so-called stack effect-pumping radon from the ground to the high floors-tends to increase gas concentration at the first floor (CV1SA).

To investigate the behavior of indoor radon with respect to the internal temperature of the room, we plot in Figure 9b the average radon concentration of CV6SA computed on a monthly basis. Both daytime/nighttime radon and their difference tend to decrease with the increasing temperature inside the apartment. This is not only due to the variation of temperature itself but to the reduction in the effectiveness of the stack effect: in closed environments, an upward movement of gas is observed, resulting from a difference in indoor-to-outdoor air density caused in turn by the gradient of temperature. The greater the thermal difference, the greater the buoyancy force, which enhances the exhalation of gas from the soil.

**Figure 10:**
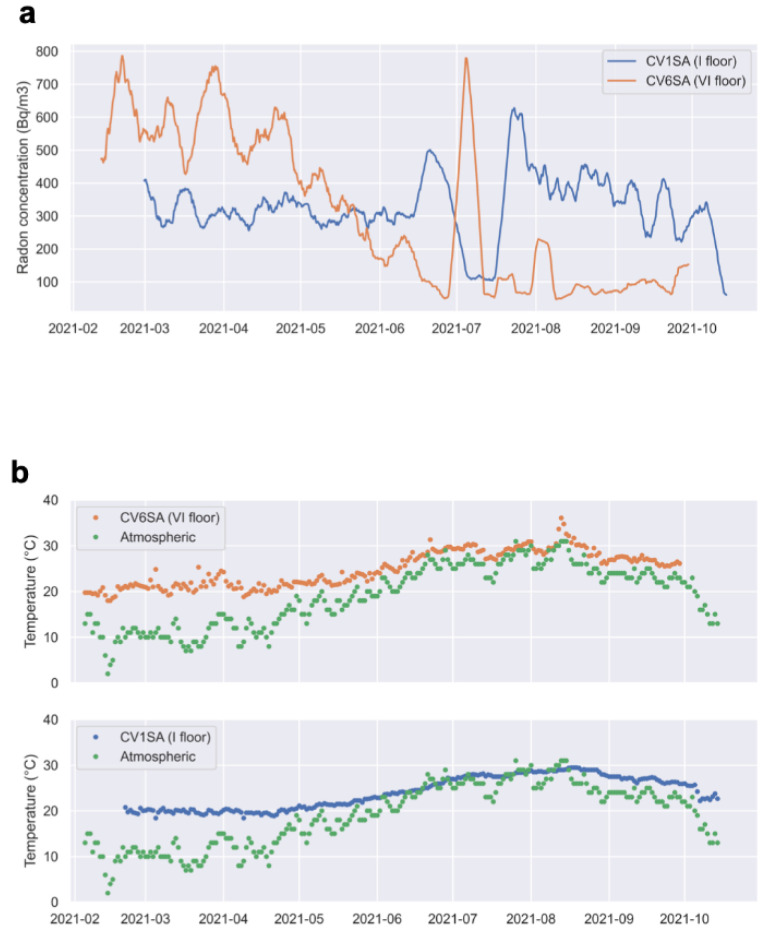
(**a**) The 1-week rolling mean of radon concentration (Bq/m^3^) measured in the rooms CV1SA, and CV6SA, on the I and VI floors (blue and orange lines, respectively). (**b**) daily temperature measured in the same rooms and atmospheric Temperature (green circles).

Figure 10a shows the 1-week rolling mean of radon concentration measured in rooms CV1SA, and CV6SA at the I and VI floors, along with (Figure 10b) the daily value of the internal temperature of each room and of the atmospheric temperature (green dots). The values of external temperature are provided by the weather forecast website https://www.ilmeteo.it (accessed on 24 November 2021 ). The higher radon level at the VI floor from February to June indicates that during the cold season, the stack effect pushes the radon gas towards the high floors (in this case, the VI), thus lowering the concentration at the first floor (CV1SA). Starting from June, the gradient of temperature between the inside and the outside gradually decreases, and the VI floor attracts far less radon, which, as a consequence, tends to stagnate at the I floor, increasing the concentration in room CV1SA. Around the middle of June, we assist in a transition on the way radon gas enters the building: at the first floor, the curve of internal temperature intersects the curve of atmospheric temperature in mid-June, and this corresponds to a peak of indoor radon concentration. The radon peak at the VI floor, after the gradual decrease that started in February, is shifted at the end of June-beginning of July, again corresponding to the intersection of indoor and outdoor temperature for this apartment.

## 4. Discussion

The experiments conducted within our pilot study on residential radon in the city of Rome reveal intermediate-to-high concentrations of indoor radon in most of the houses monitored. They are taking into account only the measurements carried out in wintertime (e.g., the ones described in Figure 3 and Table 1, because they can be considered as upper limits, for a total of 30 rooms, we observe radon levels in excess of 100 Bq/m^3^ in 18 (60%) and the reference level of 300 Bq/m^3^ is overcome in 17% of the cases. The risk of indoor radon exposure for the residents is, therefore, significant and needs to be properly assessed.

Active monitoring allows to reveal of daily and seasonal fluctuations in indoor radon concentration that are invisible to passive instruments. Diurnal/nocturnal variations, widely observed in our database, are particularly important for two reasons. First, during the night, radon tends to reach higher concentrations than during the day because of the larger thermal gradient between the indoor and outdoor, even more, pronounced in the cold season. Second, the night is the time when people are most likely to be at home. Thus exposure is enhanced, raising the entity of risk. Assuming that nighttime ranges between 9 pm and 9 am, we observe that the concentration of night radon exceeds that of day radon in the majority of cases (87%), and, more importantly, it overcomes the thresholds of 100 and 300 Bq/m^3^ in 20% and 76% of the cases, respectively, with a significant increase compared to the radon averaged over 24 h.

While in Experiment 1, the recording time of our detectors was limited to some weeks, Experiment 2 provided us with radon time series 7-month long, which allowed us to appreciate time variations on a longer timescale. Comparing the seasonal behavior of indoor radon in two apartments of the same property, the passage from the cold to the warm season is accompanied by a marked decrease in radon concentration at the VI floor and a concurrent slight decrease at the I floor, even more, evident in the frequency domain. On the one hand, this proves a remarkable variability in the behavior of this gas, strongly dependent on the internal temperature of the room and on the difference with respect to the outside temperature. On the other hand, the opposite seasonal trend shed light on the mechanism of radon transport inside the building, pumped from the ground to the elevated floors by the stack effect in closed environments.

This may be one of the reasons why our data do not fully confirm the general belief that high radon levels are associated just with the basement and the ground floor: we observe, in fact, a significant concentration of indoor radon also at the VI floor, with an average value of about 500 Bq/m^3^, and maximum as high as 1400 Bq/m^3^.

In addition to the elevation of the monitored spaces, other characteristics of a building that may affect indoor radon are age, construction materials, and isolation from the ground. Plotting the spatial distribution of average indoor radon on a geographical map, high radon values appear to cluster in the historic center, where only two of the dwellings monitored can be considered “safe.” This likely reflects the age of buildings in this area of Rome, mostly built before 1900, and consequently with construction materials largely made of tuff.

Along with the environmental conditions, anthropogenic factors such as life habits (hours of presence at home), use of heating systems, and employing natural or mechanical ventilation are decisive for the assessment of residential radon risk, as demonstrated by the rather different levels of indoor radon observed in different rooms of the same apartment.

Finally, since geology determines the geogenic radon (radon generated in the ground/emanated from shallow soil) via the radionuclide content of the outcropping rocks, the possible correlation between outcropping soils and indoor radon levels have been investigated. We found that despite the majority of the buildings under study lay over deposits of volcanic origin, the observed differences in the radon concentrations measured in the building of the same typology or in apartments at distinct floors of the same building cannot be entirely ascribed to the spatial distribution of radon emanation from underground and rather to any of the local characteristics of houses and/or anthropogenic factors described.

## 5. Conclusions

We have conducted a pilot study on residential radon in the city of Rome. Unlike the majority of the indoor air monitoring campaigns, based on passive measurement techniques, we employed commercial active radon detectors properly calibrated and intercompared. This allows us to continuously measure indoor radon concentration with accuracy comparable to that of devices specifically designed for scientific applications. Our main focus was the exploration of the range of possibilities offered by active monitoring techniques; among them, the opportunity to distinguish and compare radon levels recorded during daytime and nighttime and to reveal how seasonal fluctuations affect the monitored spaces.

Despite the limitations connected with dealing with environments such as homes, far from the controlled conditions that can be reached in the laboratory, our results might have non-negligible implications on the assessment of radon risk for residents. They can provide some preliminary indications on the sources of radon that can and cannot be controlled. The identification of the risk factors related to lifestyle habits (time spent at home, use of heating and ventilation), housing characteristics (story level, age, construction materials), and geology are useful for awareness of this issue and taking action to reduce radon in homes when the concentrations are above the WHO’s guidelines. While this is just an explorative study, leading to mostly qualitative considerations, the next step will be to design experiments aimed at quantifying the extent to which these different factors manage to explain the variability of indoor radon concentration.

## Figures and Tables

**Table 1:** Descriptive statistics of the time series of radon concentration recorded in the five rooms of Experiment 2. The values refer to the time interval March–May 2021, when all the radon devices were operating simultaneously. Columns I–III show the mean, minimum and maximum value of radon concentration (Bq/m^3^) for each time series; column IV the standard deviation, columns V–VI the number of collected data, and the length (days) of the time series.

	Mean	Min	Max	Std Dev	Count	Length
CV1SA	313.4	26.0	742.0	130.6	428	71
CV1SB	155.8	8.0	471.0	85.0	431	71
CV6SA	514.1	26.0	1414.0	306.2	429	71
CV6SB	243.0	13.0	549.0	108.4	427	71
GEA	228.1	0.0	614.0	126.9	426	71

## Data Availability

The datasets analyzed in the current study are available from the corresponding author upon reasonable request.
